# Interrater Reliability of the Canadian Occupational Performance Measure (COPM) Within Geriatric Rehabilitation

**DOI:** 10.1177/00084174251352333

**Published:** 2025-07-08

**Authors:** Dionne Plecht, Anouk van Luijk, Judith Hoek, Wilco P. Achterberg, Margot W.M. de Waal

**Keywords:** Rehabilitation, Aged, Occupational therapist, COPM, Reliability, Mots clés:, Ergothérapeute, fiabilité, mesure canadienne du rendement occupationnel (MCRO), réadaptation gériatrique

## Abstract

**Background.** The Canadian Occupational Performance Measure (COPM) is used by occupational therapists (OTs) to identify problems in the occupational performance of patients, and its use is currently expanding within geriatric rehabilitation (GR). However, due to the complexity of the target group concerns have been raised regarding consistency of administration between OTs. **Purpose.** To assess the interrater reliability of the COPM in routine GR practice. **Method.** In two GR wards with patients aged 65 years and older, two different OTs administered the COPM to the same patient. We calculated the overlap in prioritized occupational problems, as well as the intraclass correlation coefficients (ICC) of the COPM-Performance and COPM-Satisfaction scores. **Findings.** Twenty-six participants, mean age 79 (SD 7.6) with largely orthopaedic and neurological diagnoses, were assessed twice within 2–5 days (mean 3, SD 0.9). We identified a total of 355 problems, mostly in the domain selfcare (*N* = 222). For the 112 prioritized problems, two OTs had a 65% overlap. ICC values for COPM-Performance and COPM-Satisfaction were 0.566 and 0.567, respectively. **Conclusion.** In GR, the COPM has moderate IRR and a moderate percentage of overlapping prioritized occupational performance problems. Therapists should be aware of the potential scoring problem within GR and should invest in training.

## Résumé

**Description.** Les ergothérapeutes utilisent la Mesure canadienne du rendement occupationnel (MCRO) pour identifier les problèmes rencontrés par les patients en matière de rendement occupationnel. Cette mesure est de plus en plus utilisée dans le contexte de la réadaptation gériatrique (RG). Cependant, des inquiétudes ont été exprimées quant à la cohérence de l’administration entre les ergothérapeutes en raison de la complexité du groupe cible. **But.** Tester la fiabilité inter-évaluateurs de MCRO dans la pratique courante de la RG. **Méthodologie.** Dans unités gériatriques de réadaptation, deux ergothérapeutes différents ont administré le MCRO au même patient. Nous avons calculé le chevauchement des problèmes professionnels prioritaires et le coefficient de corrélation intraclasse (ICC) des mesures qui concerne le rendement et la satisfaction. **Résultats.** 26 participants, âgés en moyenne de 79 ans (SD 7·6), ont été évalués deux fois en l'espace de 2–5 jours (moyenne 3, SD 0·9). Nous avons identifié un total de 355 problèmes, principalement dans les domaines des soins personnels (N=222). Pour les 112 problèmes professionnels prioritaires, nous avons constaté un chevauchement de 65% entre les ergothérapeutes. ICC pour MCRO-rendement et MCRO-satisfaction étaient respectivement de 0·566 et 0·567. **Conséquences.** En RG, le MCRO a une fiabilité inter-évaluateurs modérée et un pourcentage modéré de problèmes prioritaires qui se chevauchent. Les thérapeutes devraient être conscients du problème potentiel de notation dans la RG et, et doivent investir dans la formation.

## Introduction

In an ageing society, the need for geriatric rehabilitation (GR) (Grund et al., 2020) is constantly rising. In the Netherlands, GR is provided by a multidisciplinary team in dedicated GR wards run by organizations specialized in long-term care, with an average length of stay of 49 days (Actiz, 2024). GR focuses on patient-centred goals, including meaningful occupations chosen by patients together with their caregivers, one of which is occupational therapy.

The Canadian Occupational Performance Measure (COPM) is a popular tool that can help occupational therapists (OTs) identify meaningful occupations by establishing an open dialogue between the patient and therapist. In a semi-structured interview setting, the therapist helps the patient to identify and prioritize occupational performance problems experienced in day-to-day occupations in the domains of selfcare, productivity, and leisure (Law et al., 2018). Assisted by the OT, the patient prioritizes the five most important occupational performance problems faced in day-to-day occupations. The patient is then asked to rate their own level of performance (COPM-P) in terms of how well they can perform the activity at that moment, and how satisfied they are with their current performance (COPM-S). A 10-point Likert scale is used for both scores. The OT subsequently shares results from the COPM with the interdisciplinary rehabilitation team (and with the patient) in order to help select appropriate treatment goals, as well as plan intervention strategies (Van Seben et al., 2017).

The COPM is widely used in the Netherlands in non-GR settings for medical specialist rehabilitation in hospitals (both in- and outpatient) and its use is expanding within GR settings. COPM measurement properties have been outlined previously, with inconsistent results (Carswel et al., 2004; Ohno et al., 2021). As patients requiring GR are characterized by greater frailty and multimorbidity compared to non-GR settings, there is a growing need for studies of COPM measurement properties particular to this specific target population. In a recent systematic literature review of COPM measurement properties within GR populations conducted by our research group (de Waal et al., 2022), we found various lines of evidence relevant to GR. One study of COPM content validity concluded that it does provide information relevant to GR (Tuntland et al., 2016). Two studies found that COPM scores are responsive to change, and are able to detect changes in the self-perception of individuals’ performance (COPM-P) and satisfaction with performance (COPM-S) over time (Edwards et al., 2007; Wressle et al., 2002). COPM scores can also be reliably assessed over time (Cup et al., 2013; Sewell et al., 2005), and interrater reliability (IRR) is moderate (Enemark Larsen et al., 2021).

Despite promising psychometric properties, the prioritizing and scoring of occupations in routine practice can still be challenging for OTs. Studies of COPM feasibility within GR found that the semi-structured nature of the instrument can lead to an unequal discussion of COPM domains, with the risk that measurements by different OTs generate different outcomes (Kjeken et al., 2004; Trentham & Dunal, 2009). This may in turn result in different patient-centred goals within GR. For example, one OT might find an occupational performance problem focused on going to the supermarket, while another OT finds that transport by bike (to the supermarket or somewhere else) is the main problem, leading to a risk of differences in goal-setting. Moreover, if COPM-P or COPM-S scores differ depending on the OT, evaluation and change scores will be biased (Mokkink et al., 2010).

Given these problems, which primarily result from the open therapist-patient dialogue format of the COPM, we wished to determine the extent of variation between OTs in everyday routine GR practice and whether there is room for improvement. We, therefore, performed a reliability study, an approach that allows important sources of variation, other than the variation between patients, to be identified (Mokkink, Eekhout et al. 2023). Therefore, the aim of this study was to test the IRR of the COPM by assessing the agreement of the five prioritized occupational performance problems, as well as COPM-P and COPM-S scores, when administered by two different OTs within a GR setting in the Netherlands.

## Methods

A multicentre clinimetric study was conducted within the University Network for the Care Sector South Holland (UNC-ZH).

### Population

The study population consisted of patients who were referred to a GR ward of two care organizations. In these organizations, it is standard procedure for all admitted patients to undergo a COPM assessment. Patients were eligible to participate if they spoke and understood the Dutch language and were aged 65 or older. Patients were excluded when they had aphasia (Law et al., 2018), a cognitive impairment that influences communication, a recent delirium (<3 months), or were clinically unstable between the first and second assessments of the COPM. The power calculation showed that a sample size of at least 26 participants was required (Eyssen et al., 2005; De Vet et al., 2011), with an anticipated intraclass correlation coefficient (ICC) of 0.7 and a 95% confidence interval (CI) of 0.2.

### Study Procedures

We anticipated recruiting all OTs working in the GR wards who assessed admitted patients and fulfilled the inclusion criteria. OTs were required to have a Dutch bachelor's degree in OT, at least one year of experience working within GR, and to have carried out regular COPM assessments for at least 6 months. The OTs enrolled patients in the study. The COPM was administered by two different OTs to the same patient, with the first assessment conducted by OTs working within the GR ward and the second conducted by the first author, who was not employed at the involved GR ward facilities. All OTs noted COPM results on the official COPM score form. OTs who conducted the first assessment received an additional form with which to record participants’ demographic data (age, sex, diagnosis, and level of education). Following the first assessment (preferably within the first week of GR), participants received a verbal description of the study as well as an information letter. Before the second assessment, participants were asked to sign a written informed consent form. Based on clinical experience and on test-retest reliability studies (Cup et al., 2013; Eyssen et al., 2005), we chose a minimum of 2 days and a maximum of 5 days between the first and second assessments. This timeframe gave potential participants sufficient opportunity to consider study participation, while minimizing changes in a participant's rehabilitation progress. The first author was blinded regarding the results of the first assessment. To determine overlap in problems identified by OTs, the first author manually matched problems prioritized in both the first and second assessments. This was carried out only after all assessments were completed. The results were then examined by two OTs working in the GR ward and any issues were discussed to reach consensus. Problems were not matched based on exact wording but by the actual meaning of a sentence. For example, “showering” was matched to “taking care of myself” and “going to the supermarket” to “doing groceries.”

### Measurement

Using the 5th edition of the Dutch COPM (Van Duijn et al., 1999), we followed the first four steps of the COPM. First, participants were asked to identify the occupational performance problems they experienced. Second, participants scored problems by importance (COPM-I) on a 10-point Likert scale (ranging from “least important” to “most important”). Third, participants were then asked to select the most important occupational performance problems they experienced, with a maximum of five. Finally, the participants were asked to score the COPM-P and COPM-S on a 10-point Likert scale (ranging from “not at all able” to “able to perform extremely well,” and from “not at all satisfied” to “extremely satisfied”). The individual COPM-P and COPM-S scores were then summed and divided by the total number of most important problems for each participant.

### Analysis

Descriptive analyses of the demographic characteristics of sex, age, education, nationality, and diagnoses of admission were carried out. To determine the percentage of overlapping problems, the total number of all reported prioritized problems of both assessments was calculated (denominator) as well as the total for overlapping prioritized problems (nominator). Mean scores were calculated for COPM-P and COPM-S. When a COPM-P and/or a COPM-S value were missing, the whole COPM was specified as missing and not included. The IRR for the mean COPM-P and COPM-S scores was assessed by calculating the ICC agreement between the first and second assessments. We first applied a one-way random effects model in order to understand whether there is any room for improvement in the measurement, given the variation between patients in our population. This was followed by a two-way random effects model, including a variance component for the rater. For both models, we calculated the ICC and the standard error of measurement (SEM) from the variance coefficients (Mokkink, Eekhout et al., 2023). The IRR was considered poor for ICC values lower than 0.41, moderate for values between 0.41 and 0.60, good for values between 0.61 and 0.81, and excellent for values higher than 0.81 (De Vet et al., 2011). Diagrams were plotted to illustrate score distributions. Descriptive analyses and diagram plotting were performed using IBM SPSS Statistics 29 (IBM Corp., 2023), and ICC analyses were performed using R (R Core Team, 2024).

### Ethics

This study was conducted according to the principles of the Declaration of Helsinki (World Medical Association, 2013) and in accordance with the Dutch Medical Treatment Contracts Act (WGBO) (Rijksoverheid, 2021). The Leiden-The Hague-Delft Medical Ethical Committee reviewed the study protocol and provided a waiver of medical ethical approval (N22·007) since the study is not subject to the Dutch Medical Research Involving Human Subjects Act (WMO). All participants were informed about anonymity, confidentiality, and the right to withdraw from the study without any repercussions. All participants were provided with a participant information letter, and oral explanations of the study and gave their written informed consent.

## Results

### Participants

A total of 26 participants were included in the study. With an average of eight patients assigned to GR each week in each of the GR wards, a total of 192 patients were potentially eligible to participate over the study period of 12 weeks. Reasons for exclusion were not registered. Table 1 shows participant characteristics. The participants were interviewed twice, each time by a different OT, with a mean interval between assessments of three days (SD 0.9, range 2–5). In total, nine OTs participated in this study, including the first author. All OTs had worked in GR rehabilitation for at least one and a half years (range 1.5–14 years). Seven of the OTs were trained in conducting the COPM and all had applied the COPM frequently over a period of at least 1.5 years (range 1.5–10 years). In one GR ward, the COPM was frequently administered by students, so these patients were not included in the study.

**Table 1:** Characteristics of the Study Population

	Participants (*n* = 26)
*Sex*	
- Females	16 (62%)
- Males	10 (38%)
*Age in years, mean, SD (min. – max.)*	79, 7.6 (66–97)
*Education* ^a^ *, %*	
- Low	10 (39%)
- Middle	12 (46%)
- High	2 (8%)
*Nationality Dutch, %*	26 (100%)
*Diagnoses for admission*	
- Orthopedic	12 (46%)
- Neurological	6 (23%)
- Lung diseases	5 (19%)
- Other	3 (12%)

^a^ Education missing *n* = 2 (8%).

### Agreement of the Five Prioritized Occupational Problems

A total of 355 occupational performance problems were mentioned, covering the three COPM domains of selfcare (222), productivity (92), and leisure (41). All participants reported occupational performance problems with selfcare (*n* = 26) and almost all with productivity (*n* = 22) in both assessments. A total of 112 prioritized occupational performance problems were reported in the first assessment, with a median of five per each individual assessment (range 2–5). In the second assessment, 125 occupational performance problems were prioritized, with a median of five per each individual assessment (range 4–5). When four occupational performance problems were prioritized in the second assessment, the number in the first assessment was always lower.

When analyzing overlap (agreement in content) between the first and second assessors, the first author's assessment was that overlap was uncertain for 16 occupational performance problems. Two independent OTs checked this selection (blinded to the first author's assessment) and agreed without any need for further discussion. The combined number of prioritized occupational performance problems from both assessments was 237, 153 of which overlapped (65.0%). Of the overlapping prioritized occupational performance problems, most were in the domain selfcare (75.2%), led by problems with personal care or functional mobility. By contrast, the domain productivity accounted for 17.3% of the prioritized occupational performance problems, mostly consisting of cooking a meal or doing household chores, with the domain leisure activities giving the fewest prioritized occupational performance problems (7.5%), mainly consisting of family visits or active recreation such as gardening or walking the dog. Regarding occupational performance problems without overlap, the majority were again in the domain selfcare (71.4%) and concerned the subdomains personal care and functional mobility. While both assessors found occupational performance problems within these domains, the content was not identical. For example, one assessor found “walking,” while another noted “going to the toilet.” Although both include movement, the nature of the activity is different so could not be matched. For other examples see Table 2.

**Table 2:** Data Examples of Five Participants, Prioritized Problems in the First and Second Assessments

	Prioritized problems in the first assessment	Prioritized problems in the second assessment	Overlap
**1**	Climbing stairs	Visit friends for coffee	No
	Washing/ironing/dusting	Walking longer distance	No
	Shopping	Shopping	Yes
	Walking	Walking with or without aid	Yes
	Washing and dressing	Washing (showering), dressing and undressing	Yes
**2**	Going to the toilet	Going to the toilet independently	Yes
	Walking indoors	Walking indoors	Yes
	Walking outside	Walking outside	Yes
	Getting in and out of bed	Washing and dressing	No
	Doing volunteer work	Going to the archive	Yes
**3**	Standing on 2 legs	Walking	No
	Make transfers	Getting in and out of bed	Yes
	Working with the animals	Taking care of animals	Yes
	Driving a car	ADL care	No
	Walking the dog	Going to toilet	No
**4**	Standing and walking	Walking	Yes
	Cold meal/sandwiches	Preparing food	Yes
	Washing and dressing	Showering	Yes
	Going to toilet	Going to toilet	Yes
		Taking care of birds	Empty
**5**	Walking	Driving mobility scooter	No
	Preparing sandwich	Morning care with partner	No
		Making coffee	Empty
		Loading and unloading the dishwasher	Empty
		Going to toilet	Empty

### Reliability of the COPM-P and COPM-S

**Figure 1.**
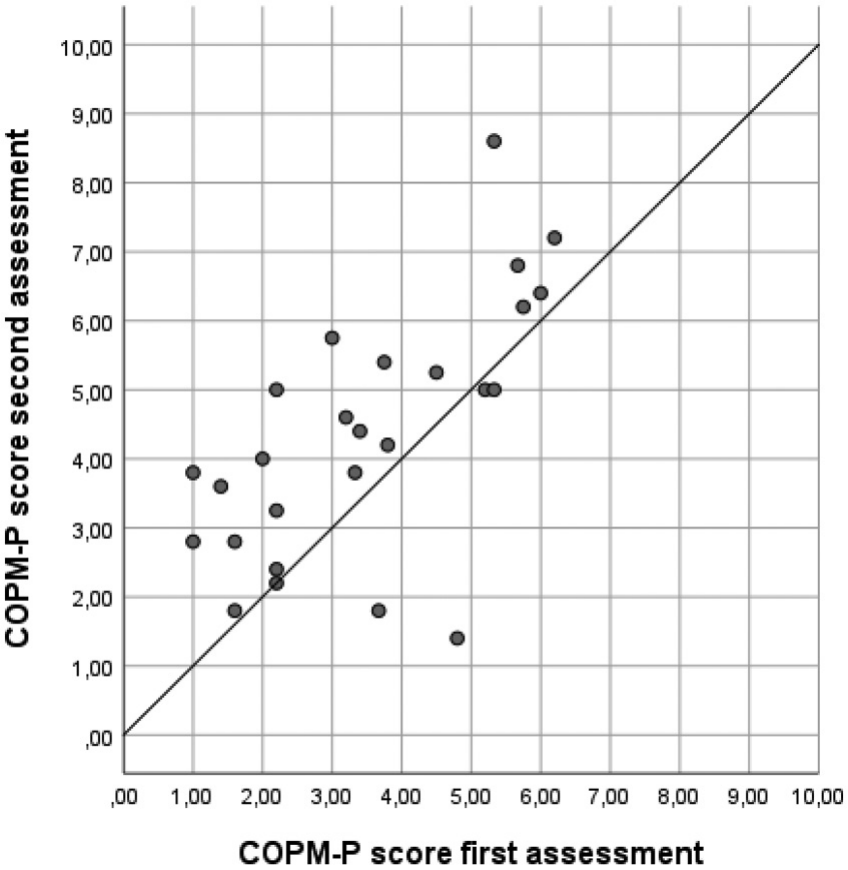
Scatter plot of the scores on Canadian Occupational Performance Measure-Performance (COPM-P) of the first and second assessments (*n* = 26).

**Figure 2.**
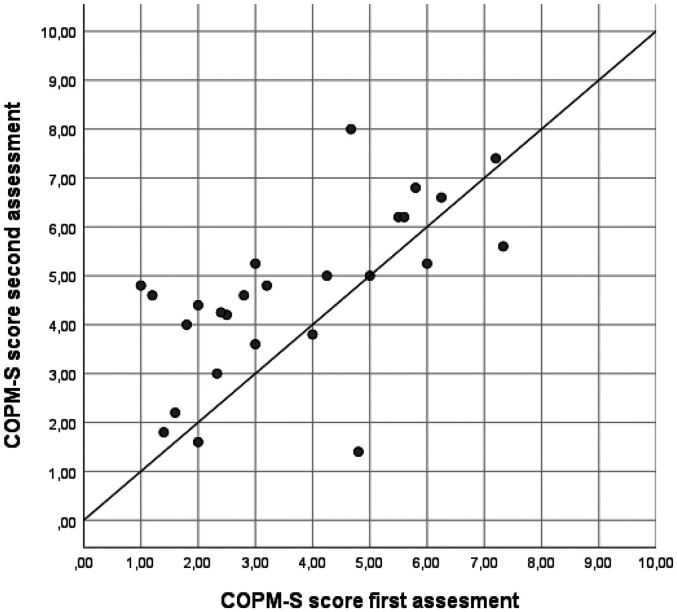
Scatter plot of the data Canadian Occupational Performance Measure-Performance-Satisfaction (COPM-S) of the first and second assessments (*n* = 26).

**Figure 3.**
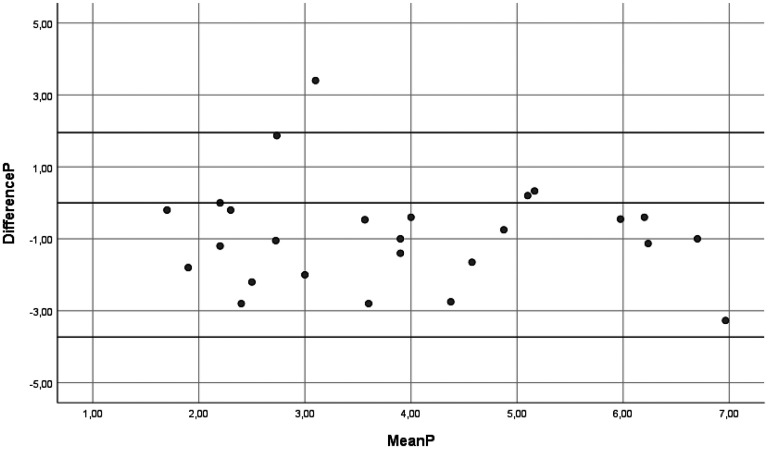
Bland Altman Plot of the mean and differences in scores of the Canadian Occupational Performance Measure-Performance (COPM-P) first and second assessments (*n* = 26). Horizontal lines represent the limits of agreement.

**Figure 4.**
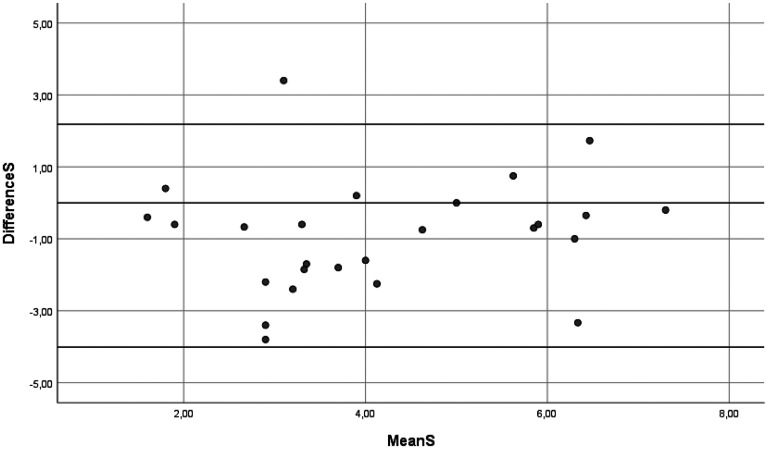
Bland-Altman plot of the mean and differences in scores of the Canadian Occupational Performance Measure-Performance-Satisfaction (COPM-S) first and second assessments (*n* = 26). Horizontal lines represent the limits of agreement.

In the two-way random models, the ICCs for mean performance and satisfaction scores were 0.566 (95% CI 0.416–0.723) and 0.567 (95% CI 0.420–0.726), respectively, with SEM values of 1.19 and 1.24, respectively. When using one-way random models, the ICCs were slightly lower and SEM slightly higher. For more details see the results in Table 3, including the variation coefficients for patients, rating, and error component. The variance components and SEM illustrate the variation between ratings, and as such highlight the room for improvement given the true variation between patients. An illustration of the correlation between mean scores on performance and satisfaction during the first and the second assessments can be found in Figures 1 and 2. The Bland-Altman plots (see Figures 3 and 4) show that scores on both COPM-P and COPM-S were lower (i.e., lower performance and lower satisfaction) in the second assessment.

**Table 3:** Results From Reliability Analyses for COPM-Performance Scores and COPM-Satisfaction Scores

Model	Mean rating 1	Mean rating 2	Var-patient	Var-rater	Var-error	ICC (95% CI)	SEM
COPM-P							
One-way random	3.47	4.36	1.81	NA	1.41	0.562 (0.416–0.723)	1.19
Two-way random	3.47	4.36	1.84	0.57	0.83	0.566 (0.420–0.726)	1.19
COPM-S							
One-way random	3.72	4.63	1.88	NA	1.62	0.537 (0.390–0.704)	1.27
Two-way random	3.72	4.63	2.02	0.47	1.07	0.567 (0.420–0.726)	1.24

Abbreviations: COPM-P = Canadian Occupational Performance Measure-Performance; COPM-S = Canadian Occupational Performance Measure-Performance-Satisfaction; SD = standard deviation; Var = variance coefficient; ICC = intraclass correlation coefficient; CI = confidence interval; SEM = standard error of measurement; NA = not applicable.

## Discussion

This study examined IRR of the COPM within GR settings in the Netherlands and found moderate overlap in identified occupational problems and moderate ICC values for both COPM-P and COMP-S scores.

### Overlap in Identified Occupational Problems

In the present study, the overlap in occupational performance problems identified by OTs was 65%, which is similar to Eyssen et al. (2005) who reported a 66% overlap (seven-day assessment interval; SD 1.6, range 4–14) in a younger group of outpatients at two hospitals (mean age 47 years). A recent study by Enemark Larsen et al. (2021) found an overlap of only 39% in a somewhat younger patient group at hospitals and medical specialist rehabilitation facilities (mean age 67, mean 10-day assessment interval; SD 3.3, range 4–18). The smaller mean assessment interval in our study (3 days; SD 0.9, range 2–5) might partly explain this difference. As our participants had little or no therapy between the first and second assessments, we expected the experienced problems to remain stable between assessments. One explanation for the moderate overlap in identified occupational performance problems may be the semi-structured character of the COPM, in combination with the older age of the participants. In a study of OTs' perspectives on identifying problems faced by older adults, Trentham and Dunal (2009) concluded that the process of identifying problems is complex due to varying cultural and cross-generational perspectives on aging. They also reported that older patients at times defer to the perceived expertise of healthcare professionals, and seem unaccustomed to identifying their own problems. However, in a more recent study conducted by Tuntland et al. (2016), 89% of participants (mean age 80.8 years) were positive regarding descriptions of their occupational performance and felt that defining these problems contributed to a closer involvement in their own rehabilitation process. In a study of COPM utility, Enemark Larsen and Carlsson (2011) stressed the need for OTs to have good interviewing skills in addition to a deep understanding of COPM concepts. This finding underlined the importance of OT confidence when administering the COPM. Indeed, several other COPM utility studies have also stressed the importance of ongoing training and support when implementing the COPM within workplace settings (Colquhoun et al., 2012; Enemark Larsen & Carlsson, 2011). In our study, almost all assessors were trained or were confident when using the COPM. However, because COPM outcomes cannot be standardized, confidence, training and experience will not eliminate all variability between OTs (Chan & Lee, 2006; Colquhoun et al., 2012).

Our study results indicated more occupational performance problems in the second assessment, which may be due to better participant understanding of their personal occupational performance problems and abilities following the first assessment. A person's own perception of their occupational performance can change over time, as well as due to changes in coping, adaptation, and recent experience or hope (Cardol et al., 2002). Therapists should thus be aware that patient knowledge concerning their personal occupational performance problems can change over time, potentially leading to changes in their prioritization of these problems.

### Interrater Reliability Values

We found moderate ICC values for scores on both the COPM-P and COPM-S. These values were higher than expected given the observed overlap in problems. Enemark Larsen et al. (2021) and Eyssen et al. (2005) also found higher ICC values than expected based on the overlap in problems in their studies. Enemark Larsen et al. (2021) explained this by stating that COPM scores can provide a reliable perception of patients’ everyday lives and representation of how patients perceive their condition at that moment, in addition to the formulation or choice of prioritized problems. Another explanation might be that some OTs ask more questions than others and thus define a more specific problem. For example, one OT might report a “selfcare in the morning” problem, while another reports the more specific problem of “dressing my upper body,” leading to scoring differences because more specific problems may be easier to score. In addition, the 10-point Likert scale might have contributed to scoring difficulties as problems with numeric scoring procedures are known to increase with age, but are also an acknowledged general problem with the COPM (Enemark Larsen et al., 2021; Eyssen et al., 2011; Kjeken et al., 2004). This supposition is supported by the most frequently heard reason for not completing the COPM: problems with scoring (Colquhoun et al., 2012; Enemark Larsen & Carlsson, 2011). This issue emphasizes the importance of providing a good explanation of scoring and underlines the need to develop strategies for overcoming the scoring problem.

### Strengths and Limitations

One of the strengths of this study was the short period between assessments. Even so, there may have been a response-shift as patients gained insight into their situation. This could have led to less overlap in identified occupational problems and less reliability of scores. Studying the consistency of the administration of the COPM is challenging, both in terms of finding the ideal research design and using the correct terminology. It could be argued that ratings can only be attributed to the client and not to the therapist, and therefore, intrarater (test-retest) reliability is examined rather than inter-rater reliability. In this study, our focus was on the variation between therapists when obtaining ratings from patients, and we therefore chose to refer to it as inter-rater reliability. Another strength was the diagnostic heterogeneity of participants, which implies that the results may be generalizable to the older population. Nevertheless, as all participants were of Dutch origin this may limit generalizability to other cultures. The study also had practical limitations. The second COPM was assessed by the same OT for each participant; therefore, the method of COPM administration used by that OT was a potential source of bias, and the data will show less variability. The same patients were not assessed by pairs of OTs in a balanced design. As a consequence, the two-way random effects models could be performed at the rater level, but not in a nested design. We therefore supplemented the analysis with one-way random effects models, which showed similar ICC and SEM values. Of the 192 potential participants only 26 were included over a period of 12 weeks. While the COPM is a standard protocol within GR wards, this relatively limited enrolment was most probably due to the fact that patients with cognitive impairment and patients assessed by student OTs had to be excluded. Therefore, we cannot say with certainty how many potential participants were not included due to exclusion criteria or because scoring the COPM-P and COPM-S was not possible. Despite this, we were able to include the required number of participants, although variation and thus confidence limits were larger than expected beforehand. Another possible limitation was that we did not pre-train therapists before data collection as in some other studies. Nonetheless, our results were comparable to Eyssen et al. (2005) and Enemark Larssen et al. (2021).

### Implications for Clinical Practice and Future Research

Since GR goals are set within week one of rehabilitation, the COPM is also administered at a time when patients are still adapting to new barriers and abilities. We know from earlier research that older individuals require more time to gain insight into their personal barriers and possibilities, so occupational performance problems may arise later within the rehabilitation process (Cardol et al., 2002). In a recent Delphi study by Pohl et al. (2020), 33 experts were tasked with developing a core set based on 119 outcome measures (not including the COPM). These experts recommended that activity measurements take place after seven days and participation measurements at twelve weeks. Based on our results and literature, we advise administering the COPM in the second week of rehabilitation, allowing patients more time to adapt to a new situation and gain more insight into their experienced occupational performance problems. However, this may be difficult in practice as there is always pressure to set goals sooner. We therefore recommend more research concerning the optimal timing of COPM administration within a GR setting, both for the patient and for the rehabilitation program. Since different OTs show only moderate levels of agreement, we recommend that the COPM (first assessment and evaluation) is administered by the same OT. In practice, if the COPM is not completed within one conversation we recommend that the same OT finishes the assessment. Studies have shown that the use of the COPM within GR has a positive influence on the participants’ involvement in their own rehabilitation process, as well as on the effectiveness of the rehabilitation itself (Eyssen et al., 2005; Tuntland et al., 2016). We recognize the challenges accompanying the scoring of COPM-P and COPM-S in practice, and OTs spend a lot of time explaining the numeric scaling. We, therefore, recommend that when scoring difficulties are encountered within GR, OTs use the first steps of the COPM to identify and prioritize occupational performance problems and only administer the numeric scale at a later stage of rehabilitation. The use of the numeric scale later in the rehabilitation process can shed light on a patient's perception of their own abilities, allowing further evaluation using the COPM. We also recommend qualitative research concerning patient and OT perspectives regarding the added value of COPM-P and COPM-S scores. Finally, we advise better training of OTs in COPM measurements,

## Conclusion

Use of the COPM in GR in the Netherlands resulted in moderate IRR and a moderate percentage of overlapping prioritized occupational performance problems. Therapists need to be aware of the scoring problems relevant to GR, and in cases where a patient has problems quantifying personal feelings, they should initially focus on identifying and prioritizing occupational performance problems.

## Key Messages

- We found that COPM scores had moderate inter-rater reliability and a moderate percentage of overlapping prioritized occupational performance problems.
- The COPM can be used reliably within geriatric rehabilitation, but therapists should be aware of challenges accompanying scoring of performance and satisfaction.
- Therapists should value training in COPM measurements, as well as frequent discussion between OTs on the use of COPM in practice.
